# Expansion of FGFR3-positive nucleus pulposus cells plays important roles in postnatal nucleus pulposus growth and regeneration

**DOI:** 10.1186/s13287-022-02903-2

**Published:** 2022-06-03

**Authors:** Meng Xu, Junlan Huang, Min Jin, Wanling Jiang, Fengtao Luo, Qiaoyan Tan, Ruobin Zhang, Xiaoqing Luo, Liang Kuang, Dali Zhang, Sen Liang, Huabing Qi, Hangang Chen, Zhenhong Ni, Nan Su, Jing Yang, Xiaolan Du, Bo Chen, Chuxia Deng, Yangli Xie, Lin Chen

**Affiliations:** 1 Laboratory of Wound Repair and Rehabilitation Medicine, State Key Laboratory of Trauma, Burns and Combined Injury, Daping Hospital, Army Medical University, Chongqing, 400042 China; 2 Department of Rehabilitation Medicine, General Hospital of Central Theater Command of Chinese People’s Liberation Army, Wuhan, China; 3 Department of Spine Surgery, Institute of Surgery Research, Daping Hospital, Army Medical University, Chongqing, China; 4 Faculty of Health Sciences, University of Macau, Macau SAR, China

**Keywords:** Intervertebral disc degeneration, Nucleus pulposus, Cell proliferation, FGFR3, Genetic mouse models

## Abstract

**Background:**

Intervertebral disc degeneration (IVDD) can cause low back pain, a major public health concern. IVDD is characterized with loss of cells especially those in nucleus pulposus (NP), due to the limited proliferative potential and regenerative ability. Few studies, however, have been carried out to investigate the in vivo proliferation events of NP cells and the cellular contribution of a specific subpopulation of NP during postnatal growth or regeneration.

**Methods:**

We generated FGFR3-3*Flag-IRES-GFP mice and crossed *FGFR3-CreERT2* mice with *Rosa26-mTmG, Rosa26-DTA* and *Rosa26-Confetti* mice, respectively, to perform inducible genetic tracing studies.

**Results:**

Expression of FGFR3 was found in the outer region of NP with co-localized expressions of proliferating markers. By fate mapping studies, FGFR3-positive (FGFR3^+^) NP cells were found proliferate from outer region to inner region of NP during postnatal growth. Clonal lineage tracing by *Confetti* mice and ablation of FGFR3^·+^ NP cells by *DTA* mice further revealed that the expansion of the FGFR3^+^ cells was required for the morphogenesis and homeostasis of postnatal NP. Moreover, in degeneration and regeneration model of mouse intervertebral disc, FGFR3^+^ NP cells underwent extensive expansion during the recovery stage.

**Conclusion:**

Our present work demonstrates that FGFR3^+^ NP cells are novel subpopulation of postnatal NP with long-existing proliferative capacity shaping the adult NP structure and participating in the homeostasis maintenance and intrinsic repair of NP. These findings may facilitate the development of new therapeutic approaches for IVD regeneration.

**Supplementary Information:**

The online version contains supplementary material available at 10.1186/s13287-022-02903-2.

## Background

Low back pain (LBP), closely related to degenerative intervertebral disc (IVD) disease, can cause not only suffering and disability of affected people, but also an enormous burden on society [1, 2]. Unfortunately, because of our limited knowledge about the etiology of IVD degeneration (IVDD), there are few effective non-surgical interventions to IVDD [3]. Discectomy and spinal fusion are thus widely utilized as a last resort.

The IVD is composed of three sub-structures including nucleus pulposus (NP), annulus fibrosus (AF) and endplate (EP). The innermost NP, rich in proteoglycans, is a gel-like tissue with great hydrostatic properties to counteract the mechanical loading on the spine. Changes in all these sub-structures are involved in disc degeneration. Among them, the NP shows the earliest and most extensive changes during the degenerative process of IVD [4, 5]. The healing ability of NP is very limited, which has been thought to be related to the hash mechanical loading microenvironment and lack of vascularity [6].

The number of NP cells decreases remarkably with age, and loss of NP cells is also recognized as an early characteristic of IVDD [7, 8]. Moreover, slow turnover of postnatal NP cells has been proved by traditional transient assays. Immunostaining of cell-cycle marker Phospho-histone H3 (PH3) revealed progressive decrease after birth and cease of the proliferation of NP cells in 4-week-old mice [9]. The continuing expansion and growth of NP tissue at a later stage has been attributed to the increased deposition of extracellular matrix (ECM) [10]. The decreased cell proliferation and quantity in aged NP is closely related to the very limited regeneration capacity of NP cells. This consensus has recently been challenged by the existence of self-renewal cells isolated from adult or even degenerated NP tissue. These cells usually express markers for mesenchymal stem cells (MSCs) [11]. A pioneering work isolated the Tie2^+^GD2^+^ NP cell population with self-renewal and multipotential abilities in subcutaneous transplantation of immunodeficient mice [12]. Notably, most of the studies are performed in cell culture in vitro. The *in situ* proliferation and contribution of these potential NP progenitors during development, growth and intrinsic repair processes following degenerative diseases and injuries have not been revealed [13].

Robust techniques including lineage tracing have recently been utilized to narrow down the gap between the in vivo observations and in vitro findings. Lineage tracing can track all progeny of the genetically labeled cells in vivo, and has recently been widely used in developmental and stem cell researches [14]. Studies using *Noto-Cre* and *Sonic hedgehog (Shh)-Cre/Shh-CreERT2* mouse lines found that the embryonic notochordal cells generate postnatal NP [15, 16]. However, there is still lack of fate-mapping studies in the field of NP morphogenesis. Many fundamental questions, including how the size and shape of an adult NP are finalized through NP cells, are insufficiently addressed.

It is increasingly appreciated that NP cells have heterogeneous cell populations. For example, there is loss of large, vacuolated notochordal-like cells and emergence of smaller, round chondrocyte-like cells in aged NP [17]. Lineage tracing study using *Krt19-CreERT2* mouse line revealed that the chondrocyte-like cells in NP tissue are aged NP cells rather than chondrocytes from surrounding tissues [18]. Moreover, a study identified that *leptin receptor (LepR)* targets notochord-derived NP cells [19]. So far, however, there is still lack of lineage tracing studies to reveal the behavior and function of NP subsets, especially the stem/progenitor cell-like population with proliferating capacity.

Fibroblast growth factor (FGF) signaling plays an important role in skeleton development and maintenance. FGF receptor 3 (FGFR3) negatively regulates the proliferation and differentiation of chondrocytes [20, 21]. Abnormal changes in IVD have been observed in mouse model mimicking human achondroplasia (ACH), a genetic skeletal disease caused by FGFR3 gain-of-function mutations [22]. Meanwhile, FGFR3 expresses in both fetal and degenerated human NP tissue [23, 24]. FGF2, a relatively specific ligand for FGFR3, has been found to promote human NP cell proliferation [25]. These data suggest that the FGFR3-expressing cells in NP may be involved in the growth and homeostasis of NP.

In this study, we found FGFR3-expressing NP cells located in the outer region of NP have superior proliferative activity. Inducible genetic tracing studies using *FGFR3-CreERT2* mice crossed with *Rosa26-mTmG, Rosa26-DTA* and *Rosa26-Confetti* mice revealed the clonal dynamics and important contribution of the *FGFR3-CreERT2-*labeled (FGFR3^+^) NP cells during postnatal growth and regeneration process.

## Methods

### Mice

FGFR3-3*Flag-IRES-GFP (*FGFR3-GFP*) mice were generated by homologous recombination in embryonic stem cells targeting an *IRES-GFP* cassette to the 18th exon of *FGFR3* locus (GFP allele). It was genotyped using primers mfgfr3-Tag-F (5’-AGCCTGAACACAGATGGCGTG-3’) and mfgfr3-Tag-R (5’-GCTTGGTCTGTGGGACTGTTG-3’), which generated a 319 bp band showing the presence of the knock-in allele. *Rosa26-mTmG* mice (Stock No.023035), *Rosa26-tdTomato* mice (Stock No.007914), *Rosa26-DTA* mice (Stock No.006331) and *Rosa26-Confetti* (Stock NO.017492) were purchased from the Jackson Laboratory. Transgenic *FGFR3-CreERT2* [26] and *Aggrecan-CreERT2* [27] mice were acquired from Prof. William Richardson (University College London, London, UK) and Prof. Jerry Feng (Texas A&M University, Texas, USA), respectively. C3H/HeJ mice were purchased from the Nanjing Model Animal Research Center. For lineage tracing, male *FGFR3-CreERT2;Rosa26-mTmG* (*R3;mTmG*), *FGFR3-CreERT2; Rosa26-tdTomato* (*R3;tdToamto*), *FGFR3-CreERT2;Rosa26-DTA* (*R3;DTA*), *FGFR3-CreERT2; Rosa26-Confetti* (*R3;Confetti*) *and Aggrecan-CreERT2;Rosa26-Confetti* (*Acan;Confetti*) mice were generated and intraperitoneally injected with Tamoxifen (Tam, 100 mg/kg, Sigma-Aldrich, T5648) in corn oil (Sigma-Aldrich, C8267) daily for 1, 3 or 5 days (Tam × 1, Tam × 3 and Tam × 5) at indicated time points. The lumbar and caudal vertebrae of each mouse, 3 ~ 5 mice per group, were dissected and collected at referential time. Discs of lumbar level (L) 2/3, L3/4, L4/5 and caudal level (C) 6/7, C7/8, C8/9 were used for analysis. All mice were maintained in the animal facility of the Daping Hospital (Chongqing, China). All animal experiments were performed according to protocols approved by the Institutional Animals Care and Use Committee of Army Medical University (Chongqing, China).

### NP cell isolation for RNA sequencing

Lumbar and caudal spines were dissected from male C3H/HeJ mice at the age of 6 ~ 8w. The surrounding ligament and muscle were removed from the vertebral column to expose the IVDs. After microdissection, lumbar (L1/2 ~ L5/6) and caudal (C6/7 ~ C10/11) NP tissues were collected and placed in separate RNA-free tubes on ice (*n* = 4 ~ 5 mice per group) [28, 29]. Total RNA was extracted using TRIzol reagent (Invitrogen, 15,596–026) according to the manufacturer’s instruction. RNA from three paired groups of caudal or lumbar NP tissues (each pair from the same group of mice) was subjected to RNA-sequencing (RNA-seq) by Shanghai Majorbio Co., LTD.

### Modified tail-looping model

We generated a modified tail-looping model [30] on *R3;mTmG* mice (male, 8w, *n* = 3 ~ 5 per group) and C3H/HeJ mice (male, 6 ~ 8w, *n* = 4 ~ 5 per group). After anaesthetization with pentobarbital sodium (100 mg/kg), mouse tails were excised from vertebrae 13. The remaining tail ends were looped and fixed to the vertebrae 5 by stainless steel wires. After 2 or 4 weeks of looping, mice in Group Loop2w and Group Loop4w were sacrificed, respectively. The unlooping surgery was performed at the 2-week-looped mice after re-anaesthetizing to remove the fixators. The mice of Group Unloop were sacrificed 2 weeks later after the unlooping. In control group, tails were excised from the same vertebrae 13 without looping as sham surgery. For each group, discs of C8/9, C9/10 and C10/11 were harvested for further analysis.

### Histology and immunostaining

Tissues were fixed in 4% paraformaldehyde and then decalcified by 15% EDTA at 4˚C. After infiltration with 30% sucrose overnight, tissues were sectioned at 10 µm thickness (CM3050 S, Leica). Sections of middle coronal or horizontal IVD tissue with the largest NP area were selected for following studies. Safranin O/Fast green staining was conducted as previously described [31]. Immunofluorescence staining was performed using a standard protocol. Sections were incubated overnight at 4 °C with primary antibody anti-Ki67 (1:200, Abcam, ab15580), followed by staining with Alexa Fluor 647-conjugated secondary antibody (1:500, Invitrogen) for 1 h at 37˚C and DAPI (1:1000, Sigma-Aldrich) for 15 min at room temperature to visualize the cell nuclei.

### EdU incorporation assay and TUNEL assay

Mice received intraperitoneal injections of ethynyl deoxyuridine (EdU, 100 mg/kg, Life Technologies, E10187) six times (with an interval of every 12 h) in 3 days (EdU × 6) at the indicated times. To detect the proliferating cells, the mice were sacrificed 2 h after the last shot [32, 33]. To detect the label-retaining cells, the samples were harvested after chasing at the indicated times [33, 34]. In single-pulse strategy using EdU (EdU × 1) to label proliferating cells, the mice were sacrificed 2 h after one shot of EdU (100 mg/kg). EdU staining was performed using Click-iT EdU Alexa Fluor 647 Imaging Kit (Invitrogen, C10640) as described by manufacturer’s instructions. TUNEL assay for apoptosis using in situ Cell Death Detection Kit [Roche, 11,684,795,910 (POD)/12156792910 (TMR red)] was performed according to manufacturer instruction.

### Image acquisition and quantification

Slices were imaged by confocal fluorescence microscope (LSM880NLO, Zeiss). Image analysis including partition of the outer and inner region of NP, calculating the height and area of NP was performed using ImageJ software (Wayne Rasband, NIH). Numbers of the immunofluorescent-positive or marker-positive cells and DAPI-positive cells in outlined NP region on mid-coronal sections were quantified by ImageJ.

### Statistics analysis

Statistical analysis was performed using GraphPad Prism 7 (GraphPad Software). The values are displayed as the mean ± standard deviations (SD). Two-tailed, unpaired Student's *t* test was used for comparisons of two groups. For comparisons of multiple groups, one-way analysis of variance (ANOVA) followed by post hoc Tukey test and Two-way ANOVA with Sidak's multiple comparisons test was conducted as indicated. Values of *P* less than 0.05 were considered significant.

## Results

### NP cells in caudal IVD and in the outer region have superior proliferative activity

**Fig. 1:**
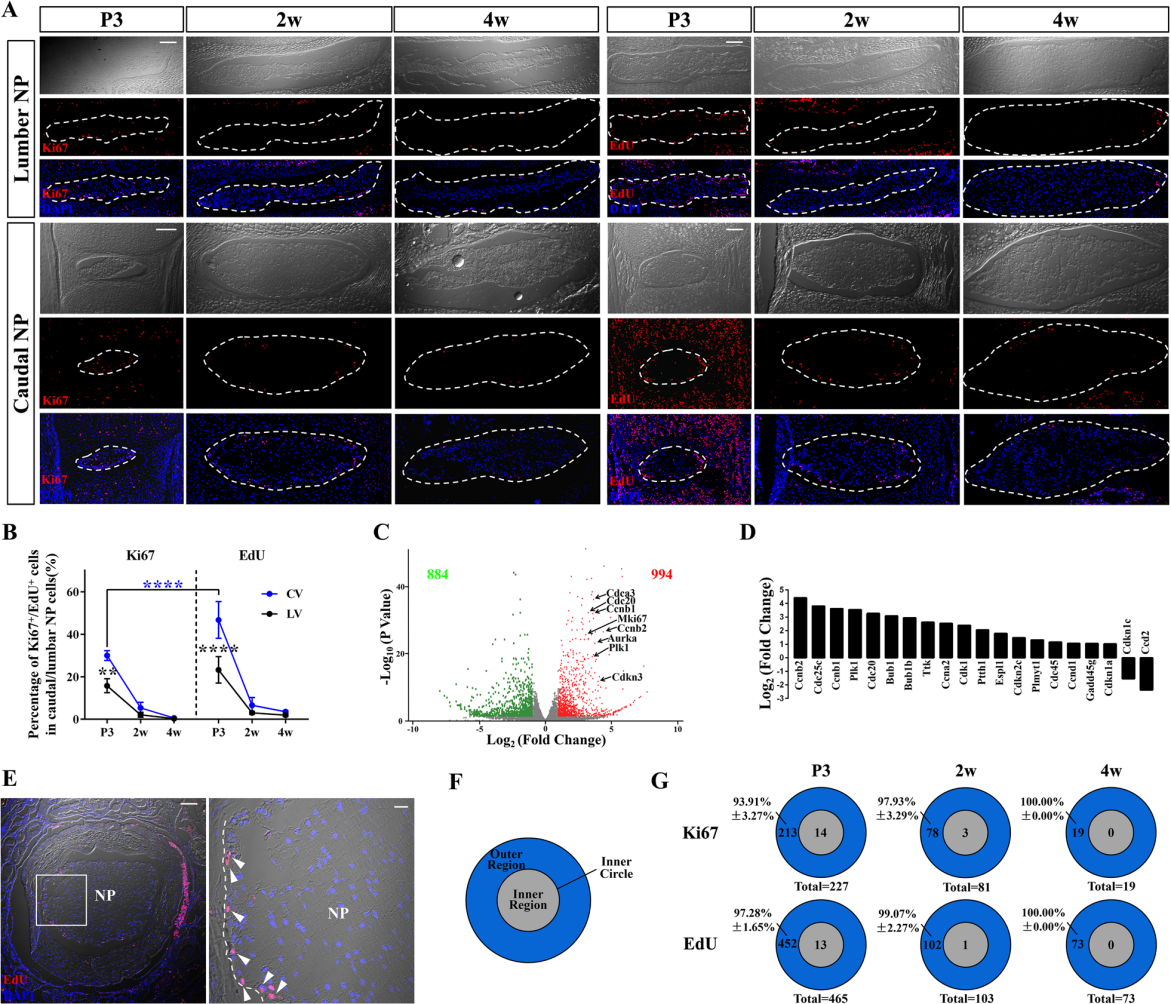
Characterization and heterogeneity of proliferating cells in postnatal NP. (**a**) Confocal microscopy images show immunostaining for Ki67 or EdU (after six-dose administration of EdU) on coronal sections of lumbar and caudal NP (dotted circle) at different ages early after birth. Scale bars, 100 µm. (**b**) Quantification of the percentages of Ki67^+^ or EdU^+^ NP cells over total NP cells in caudal vertebra (CV) or lumbar vertebra (LV) at each age reveals that sequential EdU administration have higher labeling efficiency than Ki67 immunostaining and caudal NP cells have higher proliferative activity than lumbar NP cells. **P < 0.01, ****P < 0.0001, Two-way ANOVA with Sidak's multiple comparisons test. (**c**) Volcano plot of differentially expressed genes (DEGs) from RNA-sequencing analysis between caudal NP and lumbar NP displays 994 significantly up-regulated genes and 884 significantly down-regulated genes based on a threshold of fold change ≥ 2 and *P* < 0.05. Up-regulation of cell cycling genes is indicated by their gene symbols on the plots. (**d**) KEGG pathway enrichment analysis of DEGs shows that cell cycling pathway is significantly up-regulated in caudal NP compared with lumbar NP. (**e**) Representative EdU staining images of horizontal sections of mouse disc reveal the location preference of the proliferating NP cells. Boxed area in the left image (scale bars, 100 µm) is shown in higher magnification in the right (scale bars, 20 µm). Arrowheads point EdU^+^ NP cells along the border between the NP and AF indicated by the dotted line. (**f**) Schema shows the partition of the outer region and inner region divided by the inner circle (defined as the concentric circle with half diameter of NP). (**g**) Ki67^+^/EdU^+^ NP cells in different regions are counted from both lumbar and caudal IVDs at each age. The percentages of the Ki67^+^/EdU^+^ NP cells in the outer region at different ages indicates that most of the proliferating NP cells are located in the outer region

Firstly, we evaluated the proliferation characteristics of NP in early postnatal mice by immunostaining of two typical proliferation markers, Ki67 and EdU (Fig. 1a). Ki67 is expressed from G1 to anaphase and commonly used to label cycling cells. The percentage of Ki67 positive (Ki67^+^) NP cells in lumbar vertebra (LV) was reduced from 15.73 ± 3.29% at postnatal day (P)3, 2.09 ± 1.17% at 2 weeks old(w) to 0.26 ± 0.28% at 4w, while this percentage in caudal vertebra (CV) was 27.65 ± 6.08%, 5.32 ± 2.63% and 0.73 ± 0.34%, respectively. EdU can label mitotic cells by incorporating into newly synthesized DNA. As one single pulse of EdU, which is usually used to label tissues with fast self-renewing ability, detected no division at 4w (Additional file 1: Fig. S1a), we prolonged the detecting time by sequential injections of EdU (EdU × 6) to identify more mitosis [32, 35]. This strategy can find 23.20 ± 6.21% (46.72 ± 8.66%) and 1.91 ± 0.59% (3.51 ± 0.47%) proliferating lumbar (caudal) NP cells at P3 and 4w, which had higher labeling efficiency than that of Ki67 immunostaining (Fig. 1b), and was used for the subsequent detection and fate mapping of proliferative NP cells in elder mice.

Staining studies also revealed more cell proliferation in caudal NP than lumbar NP (Fig. 1b). Furthermore, we isolated the two NP tissue separately for transcriptome sequencing (RNA-Seq). A total of 1878 differentially expressed genes (DEGs) were detected, with 994 up-regulated genes including proliferation-related genes in caudal NP compared with that of lumbar NP (Fig. 1c). KEGG enrichment analysis revealed significantly higher activity of the cell cycling pathway in caudal NP (Additional file 1: Fig. S1b, c, Fig. 1d). These data together suggest that caudal NP has higher detectable proliferation than those NP in lumbar discs.

Furthermore, we observed that the postnatal proliferating NP cells were enriched at the periphery of NP on coronal sections of both caudal and lumbar discs (Fig. 1a). Mid-transverse section also revealed the preferential gathering of EdU^+^ NP cells at the periphery of NP (Fig. 1e). We defined the inner circle as a concentric circle with half diameter of NP, the inside of the inner circle is the inner region (IR) and the outside is the outer region (OR) (Fig. 1f). Quantification of the proliferating cells in each region revealed that the location preference of both Ki67^+^ and EdU^+^ NP cells in OR (Fig. 1g). More than 90% proliferating NP cells were resident in OR at P3. With the rapid decrease of cell proliferation in elder NP, there were few Ki67^+^ and EdU^+^ NP cells at 4w that were nearly all restricted in OR (Fig. 1g). These results suggest a possible predominance role of the outer NP cells in the proliferation and expansion of NP during postnatal growth.

### FGFR3 can mark the proliferating NP cells in the outer region of juvenile NP

**Fig. 2:**
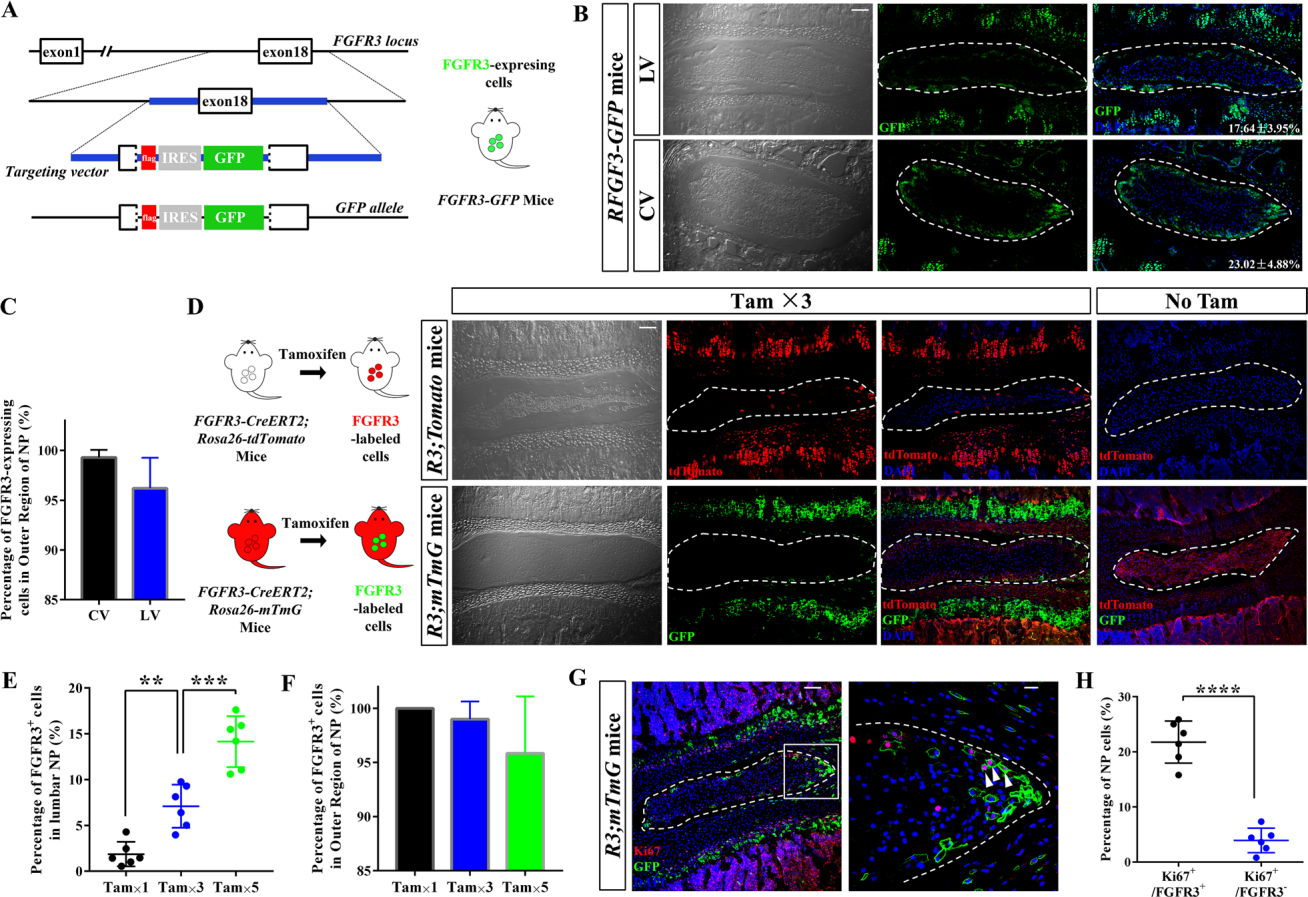
FGFR3 is expressed in the outer region of juvenile NP. (**a**) Schemas show the strategy for generation of FGFR3-3*Flag-IRES-GFP (*FGFR3-GFP*) mice with the knock-in of *GFP allele* into the 18th exon of FGFR3 to detect FGFR3-expressing cells *in-situ*. IRES, internal ribosome entry site. (**b**) Representative coronal sections of lumbar and caudal NP (dotted circle) from *FGFR3-GFP* mice at the age of 4 weeks (w) display the expression of FGFR3 at the periphery of NP. Numbers indicate the percentages of FGFR3-expressing cells over total NP cells. Scale bars, 100 µm. (**c**) Most of FGFR3-expressing cells in both lumbar and caudal NP are located in the outer region. (**d**) Representative images from *FGFR3-CreERT2;Rosa26-Tomato* (*R3;Tomato*) and *FGFR3-CreERT2;Rosa26-mTmG* (*R3;mTmG)* mice treated with Tamoxifen (Tam) or oil (No Tam) once daily for 3 consecutive days (Tam × 3) at 2w display the distribution of the *FGFR3-CreERT2*-labeled (FGFR3^+^) cells in NP (dotted circle). Scale bars, 100 µm. (**e**) Different doses of Tam (Tam × 1, Tam × 3 and Tam × 5) at 2w result in different labeling efficiency of FGFR3^+^ NP cells in *R3;mTmG* mice. **P < 0.01, ***P < 0.001, One-way ANOVA with post hoc Tukey test. (**f**) Percentages of FGFR3^+^ NP cells in outer region reveal that the location preference of FGFR3^+^ NP cells in outer region despite different doses of Tam. (**g**) Representative Ki67 immunostaining images of NP (dotted circled) from *R3;mTmG* mic with Tam × 3 at 2w are shown. Boxed area in the left image (Scale bars, 100 µm) is amplified in the right (Scale bars, 20 µm). Arrowheads indicate FGFR3^+^Ki67^+^ NP cells in cell clusters enriched at the peripheral area. (**h**) Percentage of Ki67^+^ cells in FGFR3^+^ cells is significantly higher than that of Ki67^+^ cells in FGFR3^·−^ cells. ****P < 0.0001, Unpaired Student’s* t* test

To further investigate the regional difference in NP cell proliferation, we firstly generated a *FGFR3-GFP* knock-in allele that can show the endogenous expression of *FGFR3* gene by GFP (Fig. 2a, Additional file 1: Fig. S2a). GFP expression was mainly found at the periphery of both lumbar and caudal NP in *FGFR3-GFP* mice (Fig. 2b). Quantitatively, ~ 20% NP cells were FGFR3-expressing cells and more than 95% FGFR3-expressing cells were resided in the OR of NP (Fig. 2c).

To trace the FGFR3-expressing cells genetically, we generated Tam-inducible *FGFR3* genetic lineage-tracing mice by crossing the transgenic *FGFR3-CreERT2* line with reporter alleles. Similar expressing pattern of FGFR3 at the periphery of NP was found in both *R3;mTmG* and *R3;tdToamto*)mice after Tam administration at 2w (Fig. 2d). In contrast, *R3;mTmG* and *R3*;*Tomato* mice that were injected with corn oil as controls displayed no FGFR3^+^ cells in IVDs. Then, *R3;mTmG* mice were treated with either low-, moderate- or high-dose of Tam (Tam × 1, Tam × 3 and Tam × 5 days) (Additional file  1: Fig. S2b). Higher dose of Tam resulted in increasing rate of Cre-mediated recombination (Fig. 2e). To identify and quantify cell division, sparse or single-cell labeling experiments achieved by inducing low recombination efficiency using low-dose of Tam were performed in proliferation tracing studies [36, 37]. Immunostaining of Ki67 revealed the successful labeling of the proliferating NP cells positive for FGFR3 after 3 doses of Tam applied to *R3;mTmG* mice (Fig. 2f). Quantitatively, there were significantly more Ki67^+^ cells in FGFR3^+^ than FGFR3^−^ NP cells (Fig. 2g). To analyze the proliferating behavior of FGFR3^+^ NP cells, moderate-dose of Tam was used for the next tracing study of *R3;mTmG* mice.

### Juvenile FGFR3^·+^ NP cells expand into inner region of caudal NP

**Fig. 3:**
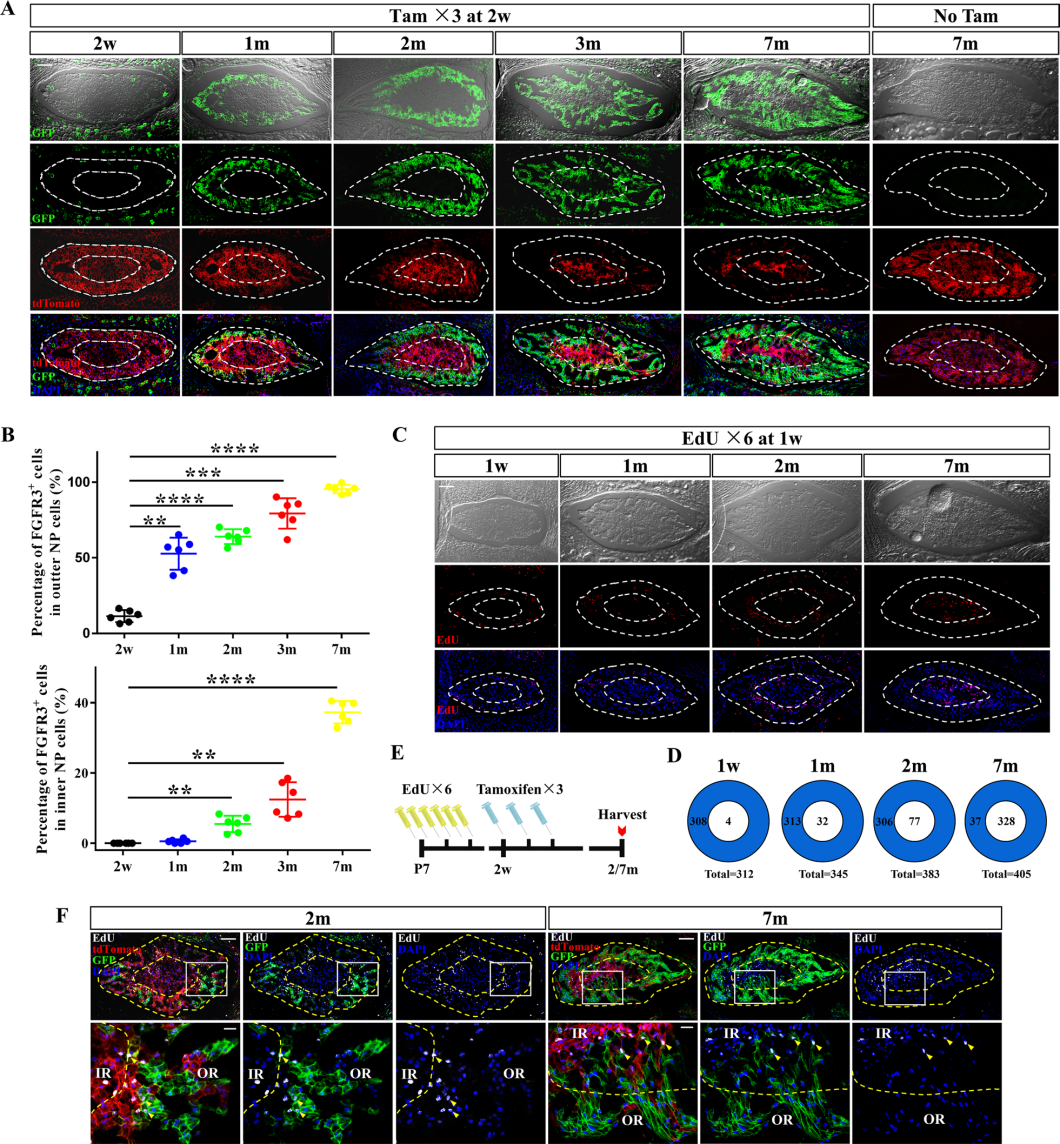
Lineage tracing reveals the expansion of juvenile FGFR3^+^ cells into the inner region of caudal NP. (**a**) *FGFR3*;*mTmG* mice were administrated with Tam at 2w and sacrificed at the different ages after chasing. Representative images show the dynamic changes of FGFR3^+^ lineage cells in caudal NP. *FGFR3*;*mTmG* mice received corn oil treatment at 2w and sacrificed at 7 m following is regarded as control (No Tam). The outer and inner region of NP is shown by the dotted boundary and inner circle of NP. Scale bars, 100 µm. (**b**) Percentages of FGFR3^+^ cells in both outer and inner region of NP increase continuously after tracing. **P < 0.01, ***P < 0.001, ****P < 0.0001, One-way ANOVA with post hoc Tukey test. (**c**) Mice were injected with EdU at 1w and sacrificed after chasing. Images at different chasing time show the positional variations of EdU^+^ cells in caudal NP. The outer and inner region of NP is shown by the dotted boundary and inner circle of NP. Scale bars, 100 µm. (**d**) Quantification of EdU^+^ NP cells in each region at different time in the EdU pulse and chase assay reveals decrease of EdU^+^ NP cells in outer region and increase of that in inner region. (**e**) Experimental design of the simultaneous tracing of FGFR3^+^ and EdU^+^ cells. *R3;mTmG* mice were administrated with EdU at 1w before receiving Tam at 2w and sacrificed at 2 m and 7 m. (**f**) Representative images of caudal NP imaged at 2 m and 7 m show that both the location shift of EdU^+^ cells and the expansion of FGFR3^+^ cells happen from outer region (annotated as OR) to inner region (annotated as IR) during chasing time. White box areas in the upper images (scale bars, 100 µm) are shown in higher magnification in the below (scale bars, 20 µm). Arrowheads indicate the colocalization of EdU^·+^ NP cells with FGFR3-lineaging cells

We next harvested the disc samples of *R3;mTmG* mice at 1 month old (m), 2 m, 3 m and 7 m after Tam administration at 2w. Abundant expansion of GFP^+^ NP cells was found preferentially at the periphery of caudal NP (Fig. 3a). Percentages of GFP^+^ cells in the OR and IR of caudal NP were calculated at different ages (Fig. 3b). GFP^+^ cells were presented in the IR of NP after 6 weeks of chasing (~ 5% inner NP cells were GFP^+^). As the tracing period extended, nearly 95% NP cells in the OR were FGFR3-lineage cells (including *FGFR3-CreERT2*-labeled FGFR3^+^ cells and their descendants) and more than 30% NP cells in the IR became FGFR3-lineage cells at 7 m. These results indicate that NP cells in the OR can derive part of the cells in the IR during the postnatal development, in which FGFR3^+^ NP cells play an important role.

To trace the proliferating NP cells, we also performed EdU retention assay (pulse and chase) by EdU pulsing at early postnatal stage (1w) and sampling at 1 m, 2 m and 7 m, respectively (Fig. 3c). Numbers of the EdU^+^ cells in different regions of caudal NP from 1w to 7 m were quantified (Fig. 3d). Because of the rapid cell proliferation and growth rate of NP at 1w [9], the EdU^+^ NP cells were found in large quantities and mainly located in the OR after pulse. After chasing, however, the number of EdU^+^ NP cells gradually declined in the OR and increased in the IR. Outer NP cells were divided more frequently (1w: 308 of 312 EdU^+^ cells in the OR) but retained lower levels of EdU (7 m: 37 of 405 EdU^+^ cells in the OR). The shift of the location of EdU^+^ cells from OR to IR suggests that the progeny of the dividing outer cells are present in the IR.

Then, we performed lineage tracing of FGFR3^+^ cells and EdU^+^ cells in caudal NP. *R3;mTmG* mice were injected with EdU at 1w before Tam administration at 2w and sacrificed at 2 m and 7 m (Fig. 3e). EdU^+^FGFR3^+^ cells, the dividing FGFR3^+^ NP cells at early postnatal stage (1w) located in the OR, were found resided around the inner circle at 2 m and into the IR at 7 m after tracing (Fig. 3f). Based on these observations, we speculate that FGFR3^+^ proliferating NP cells in the OR play important roles in giving rise to the cells in both OR and IR, resulting in the appositional growth of NP.

### FGFR3^·+^ NP cells contribute to the postnatal growth of NP

During the postnatal growth, the size (area, height) and cell number of NP were increased significantly (Additional file 1: Fig. S3a). In *R3;mTmG* mice that received Tam at 2w, the increasing quantity of FGFR3^+^ NP cells is close to that of total NP cells from 2w to 2 m (Additional file 1: Fig. S3b). No significant changes in cell death were found during this period (Additional file 1: Fig. S3c).

**Fig. 4:**
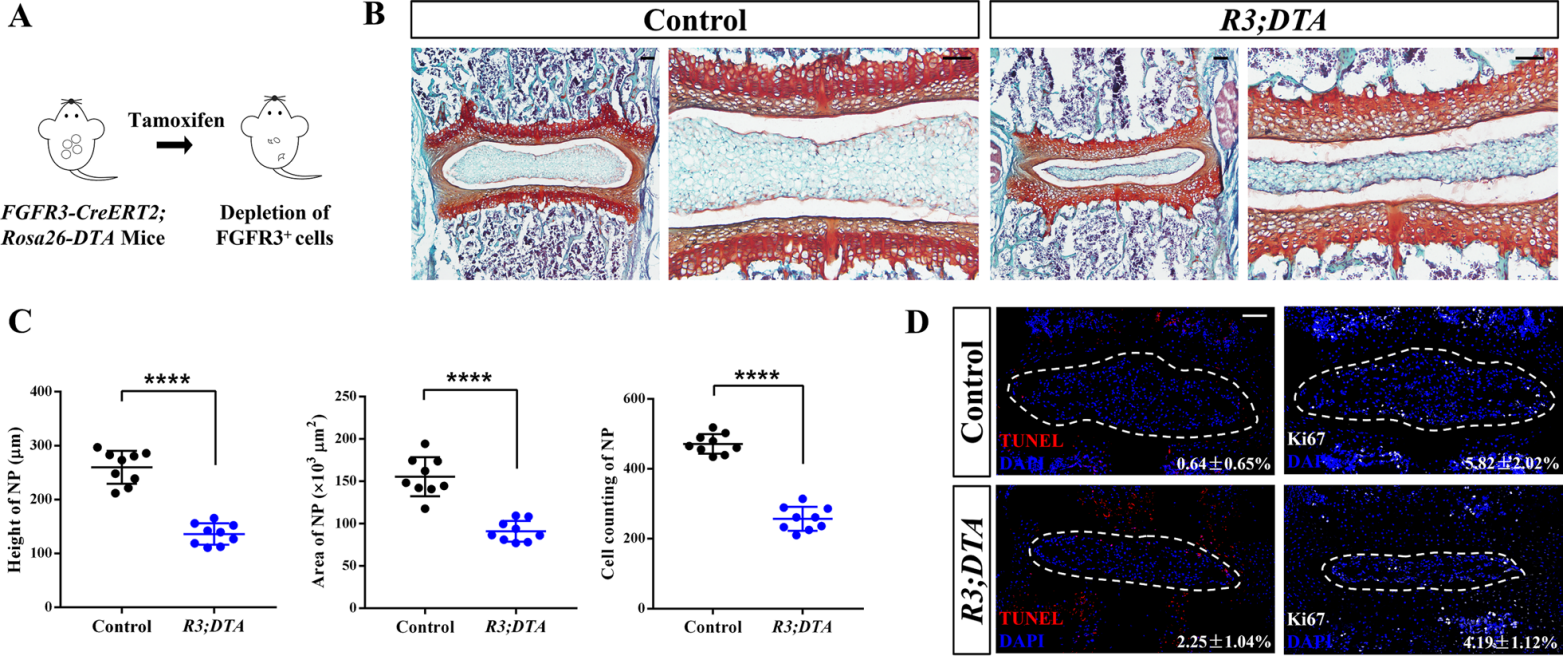
Ablation of FGFR3^+^ cells inhibits the postnatal development of NP tissue. (**a**) Schema shows the depletion of the FGFR3^+^ cells by Tam administration in *FGFR3-CreERT2;Rosa26-DTA* (*R3;DTA)* mice. (**b**) Representative Safranin O/Fast green staining images reveal pathological changes in IVD from *R3;DTA* mice induced with Tam at P7 and sacrificed at 2w. Scale bars, 100 µm. (**c**) Height, area and cell counting of NP of *R3;DTA* mice compared with the control *FGFR3-CreERT2* after Tam administration are compared, respectively. ****P < 0.0001, Unpaired Student’s* t* test. (**d**) TUNEL assay and Ki67 immunostaining were performed to detect the changes of death and proliferation of NP (dotted circle) cells after FGFR3*P*^·+^ cell depletion. Numbers indicate the percentages of TUNEL and Ki67 positive cells over total NP cells. Scale bars, 100 µm

To further verify the role of FGFR3^+^ cells in the postnatal NP growth, we bred *R3;DTA* mice by crossing *FGFR3-CreERT2* mice with *Rosa26-DTA* mice. *R3;DTA* mice and *FGFR3-CreERT2* mice (Control) received a dose of Tam at P7, separately. Spine samples were harvested 1 week later. Depletion of FGFR3^+^ cells resulted in abnormal morphology of the disc (Fig. 4a). There was a remarkable reduction of the height, area and cell quantity of NP (Fig. 4b). TUNEL assay and Ki67-IF staining revealed slightly increased cell apoptosis and decreased cell proliferation in *R3;DTA* mice after ablation (Fig. 4d). Taking together, these data indicate that the expansion of FGFR3^+^ proliferating NP cells is indispensable for the early postnatal development of NP.

### Multicolor clonal analysis reveals a unique growth pattern of postnatal NP cells

**Fig. 5:**
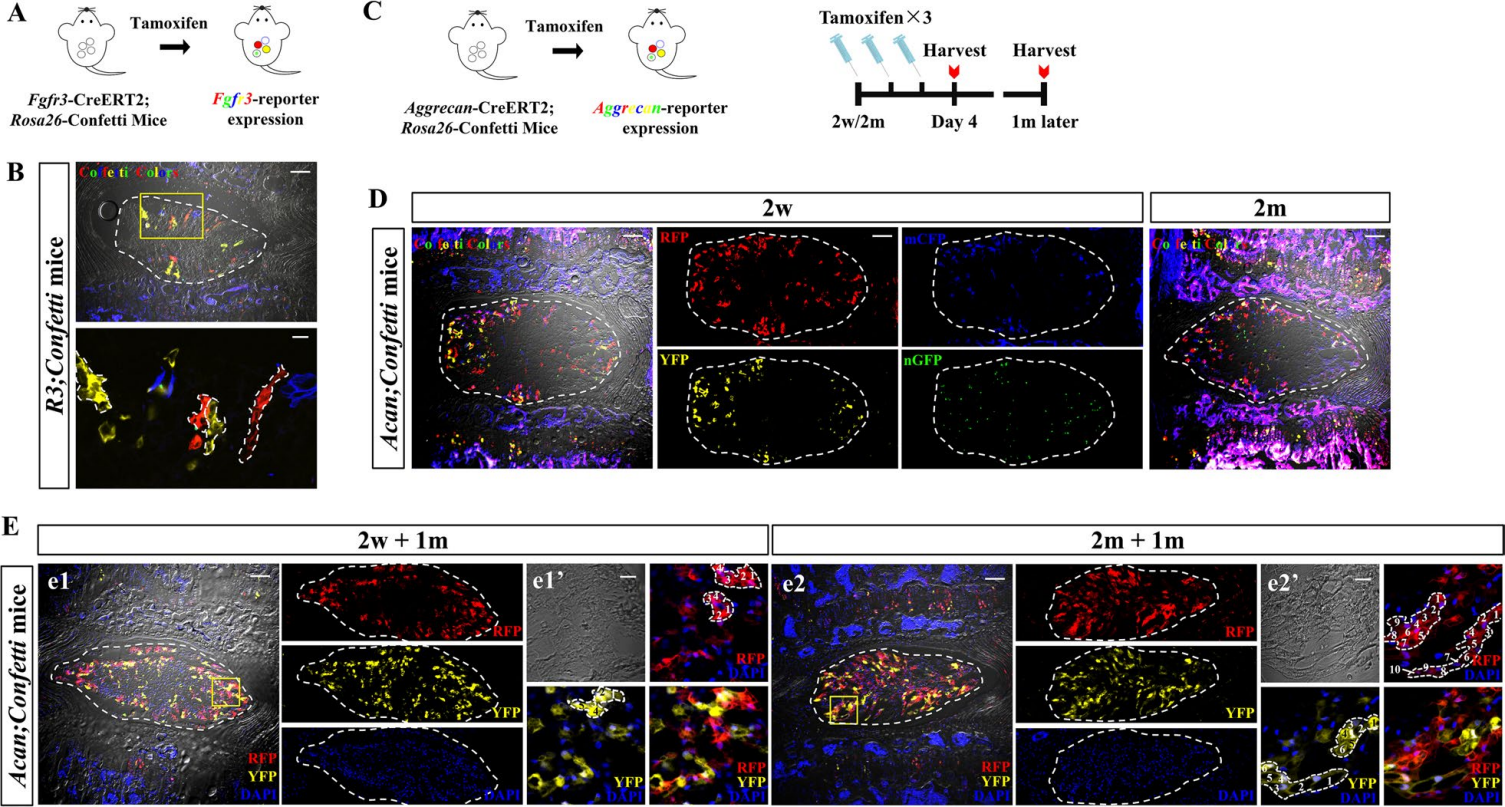
Clonal analysis by Confetti system reveals a unique growth pattern of postnatal NP cells. (**a**) Schema shows *FGFR3-CreERT2;Rosa26-Confetti* (*FGFR3;Confetti*) mice following Tam induction to achieve multicolor labeling. (**b**) Representative images from *FGFR3;Confetti* mice analyzed at 4 m after low-dose Tam injection (Tam × 1) at P7 are shown. Single-colored cell columns are found arranged along the radius axis of NP (dotted circle). Yellow box areas in the images (scale bars, 100 µm) are amplified below (scale bars, 20 µm). Typical columns are outlined in the amplified images. (**c**) Schemas show multicolor lineage-tracing strategy of *Aggrecan-CreERT2*;*Rosa26-Confetti* (*Acan;Confetti*) mice used to confirm the postnatal clonal pattern of NP cells. (**d**) Representative images show that massive NP cells are labeled with different colors in *Acan;Confetti* mice after Tam-induced recombination at both 2w and 2 m. Individual channels (RFP, mCFP, YFP, nGFP) are shown on the right of the merged image from 2w-pulsed *Acan;Confetti* mice. Scale bar, 100 µm. (**e**) After 1 m of tracing, representative images from both 2w-pulsed and 2 m-pulsed *Acan;Confetti* mice following DAPI staining are presented. RFP, YFP and DAPI channels are shown individually on the right of the merged images (e1, e2), respectively. Yellow boxed areas in (e1) and (e2) (scale bars, 100 µm) are shown at a high magnification in (e1′) and (e2′), respectively (scale bars, 20 µm). Single-colored cell clusters are found in both 2w-pulsed and 2 m-pulsed NP (dotted circle). Typical cell clusters in e1′ and e2′ are outlined, respectively. The cell numbers of each cell clusters are annotated in the dotted outlines

To further ask whether the increased FGFR3-lineage cells are directly derived from the expansion of FGFR3^+^ cells, we performed clonal lineage tracing of FGFR3^+^ cells by crossing *FGFR3-CreERT2* mice with multicolor *Rosa26-Confetti* reporter mice (Fig. 5a). The Confetti system is an optimal tool for dynamic clonal analysis. Upon administration of tamoxifen, Cre-reporter generates cells with stochastic colors in specific cellular location [cytoplasmic red (RFP), cytoplasmic yellow (YFP), membrane-bound blue (mCFP) and nuclear green cells (nGFP)], which allows the discrimination between the clonal progeny of neighboring individual cells [38, 39]. To determine the division of single FGFR3^+^ cell, we performed low-dose Tam injection (Tam × 1) at P7. After 4 m of chasing, single-colored clusters were found in NP (Fig. 5b). Interestingly, these FGFR3^+^ clones were mainly arranged into columns along the radius axis, which suggests a preference of radially orientated division or movement of NP cells during postnatal NP growth.

To know whether this clonal pattern is commonly occurred in NP cells, we next bred *Aggrecan-CreERT2* mice with *Rosa26-Confetti* mice (Fig. 5c) [40]. Aggrecan is the characteristic proteoglycan plentifully expressed by NP cells [41], and it has been reported that *Aggrecan-CreERT2;Rosa26-mTmG* mice can label nearly all the NP cells following Tam administration at both 2w and 2 m [42]. After receiving Tam at 2w or 2 m, *Acan;Confetti* mice were sacrificed at indicated time points (Fig. 5c). NP cells were labeled by multi-colors after Tam administration at different ages (Fig. 5d). After 4 weeks of chasing, single-colored cell clusters were found in both 2w-pulsed mice and 2 m-pulsed mice (Fig. 5e). We also observed typical cell columns arranged along the radius axis in the 2 m-pulsed *Acan;Confetti* mice (Fig. 5e2’). Thus, by multi-color clonal analysis, we recognized a radially orientated growth pattern of postnatal NP cells.

### Extensive expansion of FGFR3^·+^ NP cells during the recovery stage of IVD following looping protocols

**Fig. 6:**
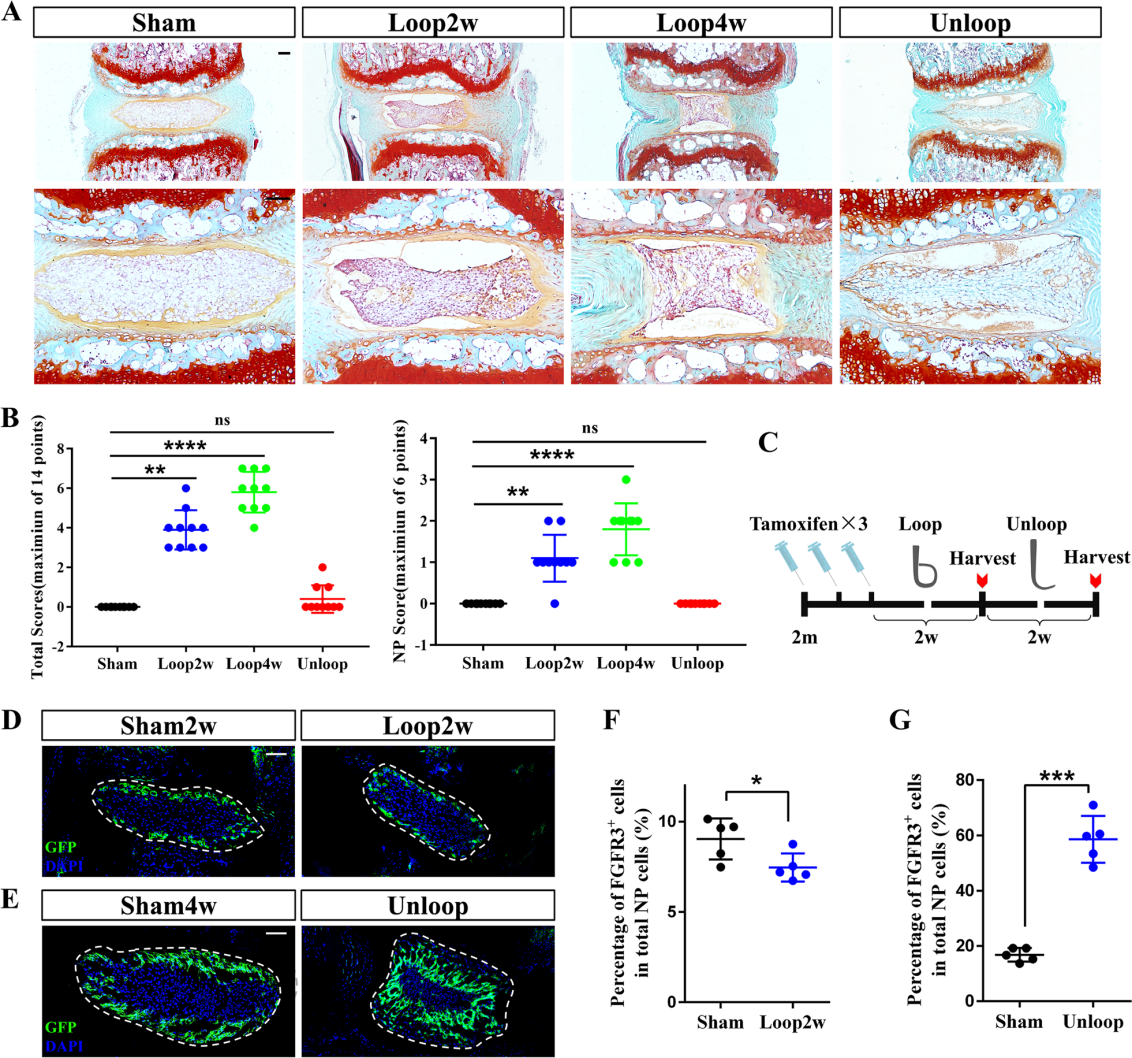
Tail-looping experiment reveals extensive expansion of FGFR3^+^ NP cells during regeneration. (**a**) Representative Safranin O/Fast Green staining images of each group of tail-looping models show the pathological changes after overloading and recovery. Scale bars, 100 µm. (**b**) Pathological scoring of NP and total IVD of each group is assessed and compared. ns = no significance, **P < 0.01, ****P < 0.0001, One-way ANOVA with post hoc Tukey test. (**c**) Experimental design shows that tail-looping model was generated using *FGFR3*;*mTmG* mice after Tam administration. (**d**, **e**) Representative NP (dotted circle) images from *FGFR3*;*mTmG* mice after looping and unlooping as well as the sham operation are shown. Scale bars, 100 µm. (**f**) The percentage of the FGFR3^+^ NP cells in total NP cells in Group Loop2w is lower than that in Group Sham2w. *P < 0.05, Unpaired Student’s* t* test. (**g**) The percentage of the FGFR3^·+^ NP cells in total NP cells in Group Unloop is significantly higher than that in Group Sham4w. ****P < 0.0001, Unpaired Student’s* t* test

Although transient immunostaining assay revealed few proliferating NP cells at 1 m, our clonal lineage tracing of NP cells at 2 m using *Acan;Confetti* mice verified the proliferating potential of adult NP cells. To further study the in vivo proliferating capacity of NP cells during the degeneration and regeneration processes, mechanical loading model by tail-looping was used [30]. Mouse tails were looped at 2 m, and tails were harvested at 2 weeks or 4 weeks after looping (Group Loop2w/Group Loop4w), or after 2 weeks tail-looping plus 2 weeks unlooping before sacrifice (Group Unloop). Mice of the control group underwent a sham surgery (Group Sham2w/Group Sham4w). Histological damages were found in discs after surgery (Fig. 6a). However, upon unlooping, the IVD underwent a visible structural restoration. Scoring analysis [43] of tail-looping model also found progressive injury of discs in looping groups, but there were no significant differences in the NP scores and the total disc scores between the Unloop and the control group (Fig. 6b).

We then applied the tail-looping model on *R3;mTmG* mice. After Tam injection at 2 m, we looped the mice immediately (Fig. 6c). After 2 weeks of chasing, there was a slight decrease of FGFR3^+^ NP cells after looping, indicating that overloading may inhibit the proliferation of FGFR3^+^ NP cells (Fig. 6d, f). However, extensive expansion of FGFR3^+^ cells in the OR of NP was found in unlooping group. (Fig. 6e, g). These results indicate that the proliferation potential of FGFR3^+^ NP cells can be induced by unloading treatment in IVD degeneration caused by mechanic loading.

**Fig. 7:**
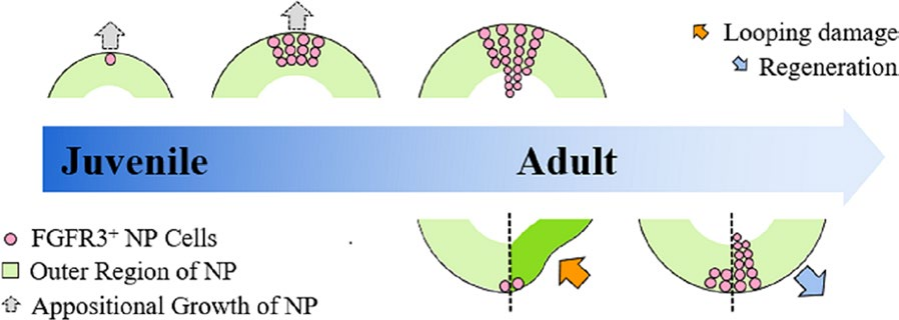
Graphical summary of the role of FGFR3^+^ NP cells in postnatal IVD growth, maintenance and regeneration. FGFR3^+^ cells are mainly found in the outer region of NP. During the early postnatal growth, FGFR3^+^ NP cells proliferate into the inner region along the radius axis of NP, which contributes to the appositional growth of NP. In tail looping and unlooping model, mechanical overloading causes pathological damage of NP. Degenerated NP can undergo intrinsic repair after removing the mechanical pressure. The expansion of FGFR3^·+^ NP cells is found to be increased significantly during the recovery stage after overloading

Briefly, in the present study, by dynamically mapping the proliferation and locations of postnatal NP cells, we revealed the in vivo proliferation characteristics of postnatal NP cells in mice and identified a novel FGFR3^+^ subpopulation of NP cells located at the outer region with superior proliferative activity. FGFR3^+^ NP cells are not only the important cell source for adult NP architecture formation during postnatal growth, but also cells participating the recovery of injured NP following mechanical loading via their expansion (Fig. 7).

## Discussion

Cell proliferation is a fundamental process that is required for the development, homeostasis, and regeneration of tissue and organs. Proliferation of postnatal NP cells, however, is revealed to be limited by detecting the expressions of proliferation markers of lumbar NP cells. Our histopathologic immunostaining and RNA-Seq studies both revealed higher proliferating capacity of caudal NP compared with lumbar NP. We propose that caudal NP is a relatively better model tissue for studying the in vivo proliferation events and patterns of mouse NP cells because of the higher detectable proliferation, faster growth rate and more round/globular configuration. From birth to around 1 week, the caudal NP is much smaller than lumbar NP (Fig. 1a). Then, the caudal NP tissue undergoes rapid global expand, while the growth of lumbar NP is accompanied by remarkable flattening of the NP cell cluster [10]. In lineage tracing experiments of FGFR3^+^ cells, we also found very limited expansion of FGFR3^+^ cells in lumbar NP without reaching into the IR of NP (Data not shown). These difference between lumbar and caudal NP may be related to their different loading environment and movement mode. NP cells respond to mechanical stimuli with changes in cell morphology, cell–cell interactions, and cell–ECM interactions [6]. Consistently, related pathways of these biological processes were found significantly up-regulated in caudal NP in our KEGG enrichment analysis (Additional file 1: Fig. S1b). Moreover, we observed that cells in the OR of NP have higher proliferative activity than that of inner NP cells. These proliferation characteristics of NP suggest that FGFR3^+^ NP cells are a novel proliferative subpopulation playing important role in postnatal development and regeneration of caudal NP.

EdU retention assay is commonly used in stem cell researches. In tissues with rapid turnover, continuing cell divisions of the EdU-labeled cells can dilute the cellular concentration of EdU to non-detectable level resulting in very few EdU^+^ cells after chasing. These cells may have slow cycling property like adult stem cells. In tissues with the relatively weak self-renewal ability such as IVD, however, the number of the EdU^+^ cells after chasing may hardly decrease or even slightly increase as these EdU^+^ cells can also be the terminally differentiated cells underwent the last duplication following the EdU administration [44]. EdU pulse and chase experiment can be also used to trace the progeny of the proliferating cells at the pulsing time [45]. As the outer NP cells serve as a proliferative population, the presence of EdU^+^ cells as well as FGFR3^+^ cells in IR after chasing indicates that the proliferation of outer NP cells can generate differentiated or quiescent inner cells. With the continuing cell expansion of the proliferating pool in the OR of NP, these NP cells finally lose their proliferative capacity during the slowdown in postnatal growth and come to reside in the IR eventually. These data reveal the cellular mechanism of the appositional growth pattern of postnatal NP and illustrate the in vivo dynamic changes of the size and architecture of postnatal NP for the first time.

As multi-color clonal analysis is able to define the clonal expansion of individual cells, we further used *R3;Confetti* and *Acan;Confetti* mice for lineage tracing and demonstrate the specific dividing patterns and further confirm the appositional growth of NP cells. We compared the clonal formation ability of juvenile and mature NP cells, and found bigger size of cell clusters in typical columnar fashion in mature NP rather than that of juvenile NP. Of note, the postnatal growth pattern of NP shared some similarities with that of articular cartilage and growth plate. Progenitor cells with self-renewal ability are found in the surface of the joints and the resting zone of growth plates [32, 46]. These progenitors can produce chondrocytes into deeper regions forming chondrocyte columns in articular cartilage and growth plate. During the postnatal growth of the mouse long bone, bigger size of chondrocyte columns is also found in elder growth plate compared with shorter columns formed in neonatal stage [45]. Therefore, our lineage tracing assay of the proliferative histories of NP cells provides valuable information for NP progenitor cells. Whether the FGFR3^+^ NP cells are kind of precursor subsets is worthy to be studied.

Since invasive injuries by puncture or incision may bring cells from AF, fascia and other surrounding tissue into NP [47], while FGFR3 is also expressed in inner AF and EP, the potential involvement of the FGFR3^+^ AF and EP cells can interfere with the explanation of results. Moreover, biomechanical overloading, a common predisposing factor of IVDD, causes loss of NP cells and impairs the function of NP to produce ECM [48]. We thus used mechanical loading model rather than puncture model to analyze the roles of FGFR3^+^ cells in IVD repairing. In the tail-looping model we used, degenerative changes of the NP tissue occur after looping. Also of note, recent studies found that loading with proper magnitude, frequency and duration can promote the production of ECM in NP [6, 49]. But whether the loading damage can stimulate NP regeneration and repair by increasing cell proliferation and differentiation of NP stem/progenitor cells has not been addressed. By looping the tail for 2 weeks with following 2 weeks of unlooping, we found that the degeneration process and following regeneration process of IVD can be to some extent mimicked. The increasing expansion of FGFR3^+^ NP cells during the unlooping recovery stage confirms the self-repair potential of the degenerated NP in mice. Besides, diverse cellular origins except the stem/progenitor cells for the regeneration of distinct adult tissues have been found recently [44]. In tissues such as liver [50], lung [51], intestine [52], bone [53] and muscle [54], during the degeneration and injury, lost cells can be replenished through cell proliferation of committed progenitors or even the differentiated cells indicating that the ability to proliferate is not only reserved in rare stem/progenitor cells. In clinical treatment of IVDD, new therapeutic options aiming to regenerate the IVD are currently under investigation. Our data suggest that FGFR3^+^ NP cells may be a source of cells for NP regeneration or even potential seed cells that can be used for future cell therapy of IVDD. However, the detailed molecular mechanism triggering the proliferation of FGFR3^+^ NP cells especially during regeneration and the possibility of using or modulating FGFR3^+^ NP cells for early prevention and treatment of IVDD need to be further elucidated.

## Conclusion

We identified a novel FGFR3^+^ subpopulation of postnatal NP that are indispensable for the NP morphogenesis and repairing. The demonstration of the long-existing proliferating potential of the FGFR3^+^ NP cells especially during degeneration and regeneration provides assertive in vivo evidence supporting the involvement of precursor-like cells in the homeostasis maintenance and intrinsic repair ability of postnatal NP.

## Supplementary Information

**Additional file 1**. Additional figures.

## Data Availability

The raw reads of our RNA-sequencing data are all available through the NCBI Sequence Read Archive (SRA, http://www.ncbi.nlm.nih.gov/sra/) under accession number PRJNA826213.

## Abbreviations

AF: Annulus fibrosus
CV: Caudal vertebra
ECM: Extracellular matrix
EdU: Ethynyl deoxyuridine
FGF: Fibroblast growth factor
FGFR3: FGF receptor 3
IR: Inner region
IVDD: Intervertebral disc degeneration
LV: Lumbar vertebra
MSC: Mesenchymal stem cell
NP: Nucleus pulposus
OR: Outer region
RNA-Seq: RNA sequencing
Tam: Tamoxifen
