# Supplementary material for: Expansion of FGFR3-positive nucleus pulposus cells plays important roles in postnatal nucleus pulposus growth and regeneration
Source: Stem Cell Res Ther. 2022 Jun 3;13:227. doi: 10.1186/s13287-022-02903-2 (PMC9166488; doi:10.1186/s13287-022-02903-2)
Supplement: Supplementary file 1 — Additional file 1. Additional figures. [file 13287_2022_2903_MOESM1_ESM.pdf]

Supplementary Information

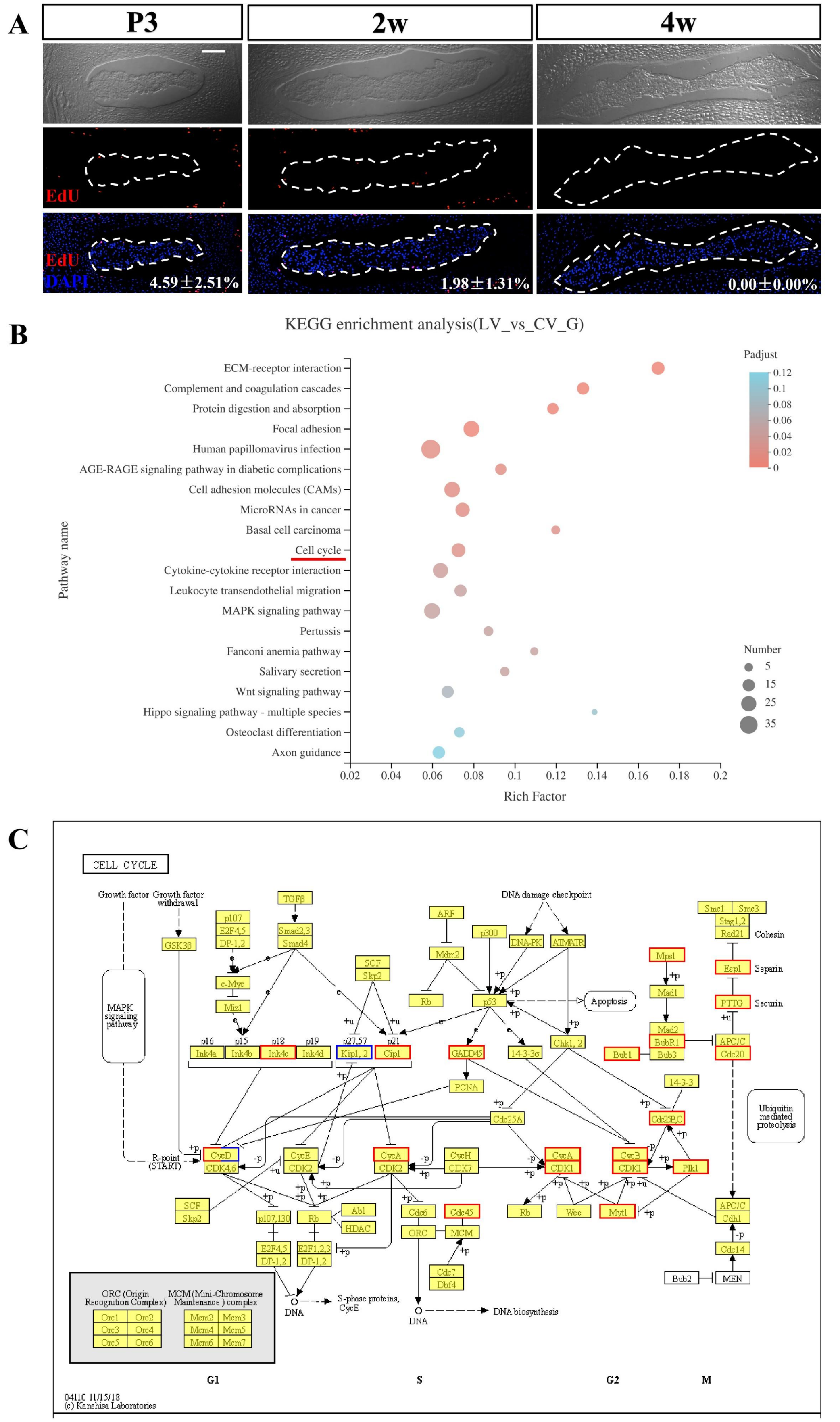

Fig. S1 **(a)** Images of EdU$^+$ proliferating cells (after one-dose of EdU) in lumbar NP (dotted circled) at different time points early after birth. Numbers indicate the percentages of EdU$^+$ NP cells over total NP cells. Scale bars, 100 μm. **(b)** KEGG pathway enrichment analysis of differentially expressied genes (DEGs) from RNA-sequencing analysis between caudal NP and lumbar NP reveals DEGs were enriched in 20 pathways including cell cycling pathway. **(c)** The detailed description of the upregulated genes in cell cycling pathway enriched by KEGG analysis.

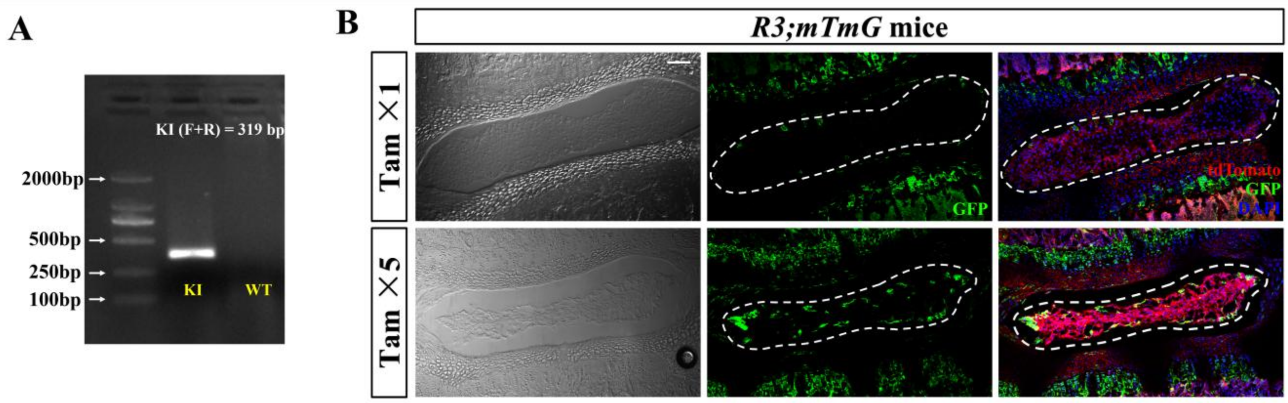

Fig.e S2 **(a)** PCR genotyping of the knock-in (KI) allele (319 bp) using mfgfr3-Tag-F and mfgfr3-Tag-R primer mix. WT, wild type. **(b)** Representative images of lumbar sections from *R3;mTmG* mice treated with low-dose of Tamoxifen (Tam×1) or high-dose of that (Tam×5). Scale bars, 100 μm.

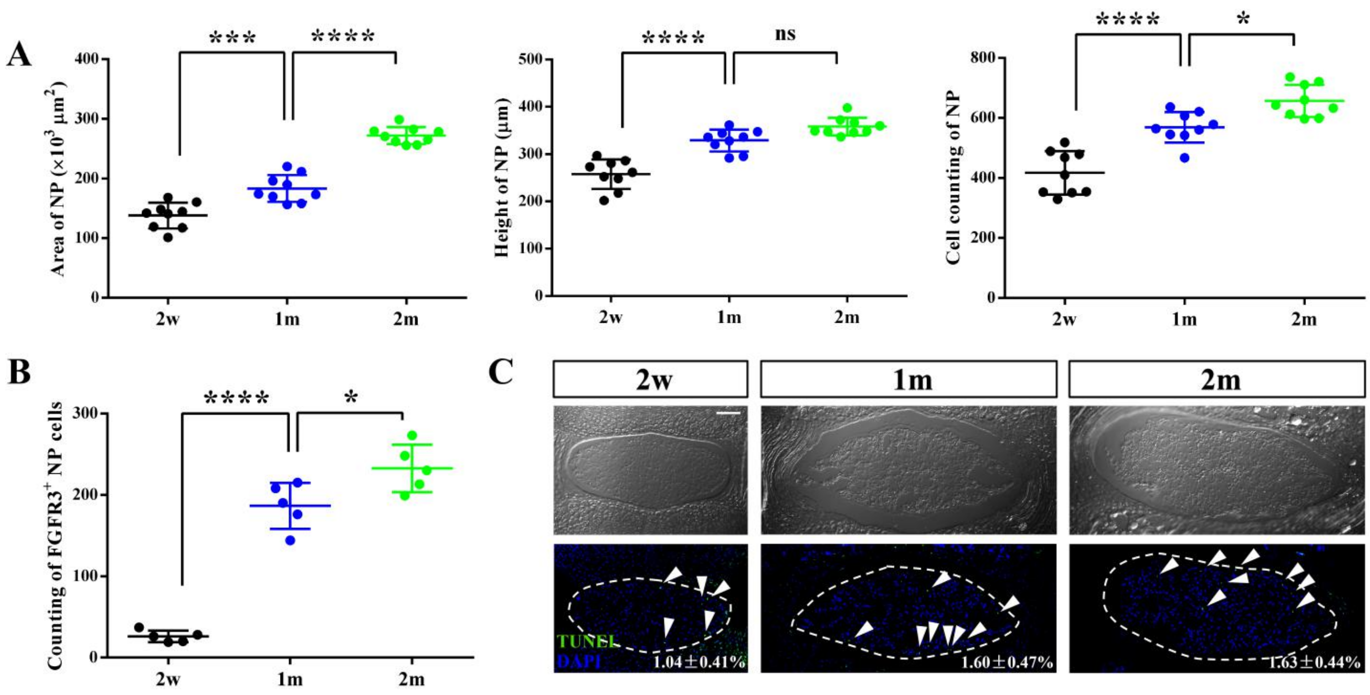

Fig. S3 **(a)** The height, area and cell quantity of NP were analyzed at 2w, 1m and 2m. ns = no significance, ** P<0.01, *** P<0.001, **** P<0.0001, One-way ANOVA with post hoc Tukey test. **(b)** Increasing FGFR3$^+$ NP cells were also found in *R3;mTmG* mice with Tam injection at 2w sacrificed at 2w, 1m and 2m. * P<0.05, **** P<0.0001, One-way ANOVA with post hoc Tukey test. **(c)** Representative TUNEL staining images of NP at 2w, 1m and 2m. Arrowheads indicate TUNEL positive cells and the numbers indicate the percentages of TUNEL positive cells over total NP cells at different ages. Scale bars, 100 μm.
